# Evaluation of Hookworm Diagnosis Techniques from Patients in Debre Elias and Sanja Districts of the Amhara Region, Ethiopia

**DOI:** 10.1155/2021/6682330

**Published:** 2021-04-28

**Authors:** Ayalew Jejaw Zeleke, Ayenew Addisu, Adane Derso, Yalewayker Tegegne, Meseret Birhanie, Tekeba Sisay, Mulugeta Aemero

**Affiliations:** ^1^Department of Medical Parasitology, School of Biomedical and Laboratory Sciences, College of Medicine and Health Sciences, University of Gondar, Gondar, Ethiopia; ^2^Institute of Biotechnology, University of Gondar, Gondar, Ethiopia

## Abstract

**Background:**

Inappropriate diagnosis could intimidate the prevention and control of hookworm infection. Thus, this study was aimed at evaluating the performance of hookworm diagnosis methods.

**Methods:**

An institution-based cross-sectional study was conducted from patients in Debre Elias and Sanja districts of the Amhara region, Ethiopia, from November 2019 to January 2020. The study subjects were selected conveniently. After the data was entered into Statistical Package for the Social Sciences (SPSS), sensitivity, specificity, predictive values, test accuracy, and agreement of the different hookworm diagnosis methods, namely, test tube flotation technique (TFT), McMaster (MM), formol-ether concentration (FEC), Kato-Katz (KK), and direct wet mount microscopy (DWMM), were calculated by using SPSS software. The composite reference standard (CRS) was used as the gold standard method. The kappa (*κ*) test was used to measure the level of agreement between diagnosis tests.

**Result:**

A total of 389 stool samples were collected from patients in the study. The overall prevalence of hookworm was 63.24%. The test tube flotation technique (TFT) was found to be the highest both in terms of sensitivity and diagnostic accuracy (100%). McMaster (MM) was the second most sensitive test (68.7%), followed by FEC (44.3%) and KK (38.2%). On the other hand, DWMM had the lowest sensitivity (37.4%), and its diagnostic accuracy was also the least (60%). Only TFT had a perfect agreement (agreement = 100%, kappa = 1) with the CRS. The sensitivity of DWMM, KK, and FEC showed a kind of linear function with the intensity of infection, but TFT and MM methods were not affected by the infection intensity.

**Conclusion:**

Hookworm is still a public health problem in the study area. TFT is by far more sensitive than MM, FEC, KK, and DWMM techniques.

## 1. Background

Hookworm is one of the big three soil-transmitted helminths (*Ascaris lumbricoides*, *Trichuris trichiura*, and hookworm). These three helminths infect more than two billion people worldwide, and the disease burden might approach that of malaria [1]. Soil-transmitted helminth infections cause a great and frequently silent burden of morbidity and mortality on poor populations in developing countries that accounts for approximately 85% of the NTD burden [2, 3]. Despite the existence of control programs, the hookworm disease burden remains high. Currently, hookworm affects approximately 500 million people, with 5.1 billion at risk for acquiring infection worldwide [4].

Hookworm is a blood-feeding intestinal worm, and the mature larvae ingest the blood, rupture the erythrocytes, and degrade the hemoglobin by attaching to the gut wall, which results in iron deficiency anemia. Hookworm changes feeding sites and during feeding secretes an anticoagulant, resulting in additional blood loss from the damaged gut wall. The most damaging effects of hookworm infections include impaired physical, intellectual, and cognitive development of children, increased mortality in pregnant women and their infants, and reduced work capacity of adolescents and adults [5, 6].

The prevention and control of hookworm infection involve many approaches like sanitary disposal of feces, early diagnosis, and chemotherapy and health education [7–10]. However, improper diagnosis and emergence of drug resistance could threaten the prevention and control of the parasite [11, 12]. Misdiagnosis of hookworm is unfortunate because a misdiagnosed patient may be given a treatment that is ineffective against the parasite, and therefore, it would not alleviate the patient's suffering or stop the progressive wasting of health [13].

There are different methods of hookworm diagnosis: conventional direct wet mount microscopy, concentration, FLOTAC, McMaster, Kato-Katz, culture, and molecular analysis [14, 15]. Direct stool microscopy is solely used in all health care service providers in Ethiopia for intestinal parasite detection including hookworm infection. It is generally believed that it has low sensitivity which might be affected by multiple factors. This indicates that direct wet mount microscopy may lead to inappropriate diagnosis of intestinal helminth infections as the amount of stool specimen examined is minimal compared to the other parasitological techniques [16]. In the FEC technique, as the amount of stool specimen examined is large, it can improve the diagnosis of hookworm [17]. Moreover, the detection rate of KK for hookworm can be improved by examining duplicate smears from a single stool specimen [15]. Therefore, for improved intestinal parasitic disease control, cost-effective, simple, sensitive, and consistent diagnosis tests are essential [18].

Although there exist different types of microscopic and a few advanced hookworm diagnosis methods [14], there are limited studies that show comparisons on their sensitivity. In this regard, an evaluation of these diagnosis techniques to identify the most sensitive method of hookworm detection is crucial. Therefore, the data generated from this study could serve as an input for decision-making among health care service providers, local health planners, and policymakers.

## 2. Methods

### 2.1. Study Design, Period, and Area

An institution-based cross-sectional study was conducted from November 2019 to January 2020 in Debre Elias and Sanja districts of the Amhara region, Ethiopia. Debre Elias is 430 km far from Addis Ababa and 310 km from Gondar Town. Sanja is located 65 km northwest of Gondar Town and 792 km away from Addis Ababa. The estimated total population of Sanja Town is 26,000. Sanja has an altitude of 1800 m above sea level with annual rainfall ranging from 800 to 1800 mm and an average temperature of 25°C to 42°C. On the other hand, Debre Elias has a total population of 82,150 [19]. There are two health institutions (one health center and one hospital) in each of the study areas. The study was conducted among adult patients who were visiting the health institutions. Study participants who took antihelminthic drugs for the last one month were excluded.

### 2.2. Study Population, Sample Size Determination, and Sampling Technique

All adult clients (patients) who came to the health institutions of Debre Elias and Sanja districts were the source population. Similarly, all adult clients (patients) who came to the health institutions during the study period were considered the study population, whereas all adult clients (patients) who come to the health institutions and also who were requested for stool microscopy and volunteered to participate during the study period were our study participants.

The sample size was determined using a formula (*Z*^2^∗*P*(1 − *P*)/*d*^2^) for estimating a single population proportion. The proportion of hookworm infection is assumed to be 50%. By considering a 95% confidence level and a 5% expected margin of error, the calculated sample size was 384. To minimize errors arising from the probable occurrence of noncompliance, 5% of the sample size was added to the calculated sample size, thereby increasing the sample size to about 400. The study subjects were conveniently selected during the study period. An equal number of study participants were taken from each district.

### 2.3. Study Variables

The outcome variable was the positivity rate (prevalence) of hookworm infection, while gender and hookworm diagnosis methods were the independent variables.

### 2.4. Laboratory Works

#### 2.4.1. Stool Sample Collection

Prior to the laboratory investigation, a well-structured questionnaire was used to collect sociodemographic and other characteristics of the study participants. A clean, dry, and leak-proof container was used to collect about 20 g stool specimens. Then, the stool samples were processed following five types of microscopic stool examination methods at the health facilities.

#### 2.4.2. Microscopic Examinations of Stool

All specimens were investigated using direct microscopy, Kato-Katz, formol-ether concentration, McMaster, and test tube-cover slide flotation methods following the protocols described before [10, 20].

*(1) Direct Wet Mount Microscopy*. A fresh stool sample (about 2 mg of stool) was placed on two slides with a wooden applicator stick, emulsified with a drop of physiological saline (0.85%), covered with cover slides, and examined under a microscope using a 10x objective.

*(2) Formol-Ether Concentration*. This test was performed by mixing thoroughly around 1 g of feces in 3-4 ml of 10% formaldehyde in a glass container. Two layers of gauze were placed in a funnel, and the contents were strained into a 15 ml centrifuge tube. Then, additional 3 ml of 10% formaldehyde and 3 ml of ether were added. The solution was mixed well and centrifuged at 1000 revolution for 3 min. The supernatant was removed, and two slides were prepared from the sediment and finally examined with a 10x objective of the microscope.

*(3) Kato-Katz Technique*. It was performed by transferring the sieved stool to the templates which deliver 41.7 mg of stool. The stool was covered with cellophane which was previously immersed with malachite green. Identification and quantification of the ova were done. Eggs counted per slide were multiplied by 24 to convert into the number of eggs per gram of stool (EPG). The parasite load or intensity was defined as light, moderate, and heavy according to the World Health Organization (WHO) guideline [21]. Two Kato slides were prepared from each sample.

*(4) McMaster*. It was performed by mixing 2 grams of stool with 30 ml of flotation solution (saturated sodium chloride solution at room temperature, density ~ 1.20). The fecal suspension was poured through a wire mesh to remove large debris. Then, 0.5 ml aliquot was added to each of the two chambers of a McMaster slide. Both chambers were examined under a light microscope using a 10x objective, and the fecal egg count was expressed as eggs per gram of stool (EPG) and was obtained by multiplying the total number of eggs by 50 [22].

*(5) Test Tube Flotation*. It was performed by mixing 2 grams of stool with 30 ml of flotation solution 2 (saturated sodium chloride solution at room temperature, density ~ 1.20). The fecal suspension was poured into a 10 ml test tube through a wire mesh to remove large debris. Then, a cover slide was placed to the top of the test tube. This allowed the ova of the parasite to adhere to the cover slide. Finally, the cover slides were placed under a light microscope and examined using a 10x objective.

### 2.5. Quality Assurance Mechanisms

To avoid observer bias, two experienced laboratory personnel performed the microscopic examination of the slide smears blindly and independently. Independent readings of slides by the laboratory personnel were checked by another expert. The results of their observation were recorded for later comparison on separate sheets. Quality control was done by repeating all discordant results.

### 2.6. Data Analysis

Data was directly entered into SPSS software. It was also checked for its completeness, and finally, it was analyzed using the software. To estimate the sensitivity, specificity, PPV, and NPV, the standard formulas were used: (i) sensitivity = TP/(TP + FN) × 100%; (ii) specificity = TN/(TN + FP) × 100%; (iii) PPV = TP/(TP + FP) × 100%; and (iv) NPV = TN/TN + FN) × 100%. The kappa (*κ*) test was used to measure the level of agreement among the tests. A *κ* value of 0.2–0.60 represents a fair to moderate agreement, a *κ* value of 0.60–0.80 represents a substantial agreement beyond chance, and a *κ* value of >0.80 represents almost perfect agreement beyond chance [23].

## 3. Result

### 3.1. Hookworm Prevalence Rate by Diagnosis Techniques

A total of 389 study participants were enrolled in the study. Out of these, 221 (56.8%) were males and 168 (43.2%) were females. The mean age of the study participants was 32.9 ± 13.78 years, and most (88.4%) of them were from 18 to 45 years of age. Out of 389 patients, who were subjected to intestinal parasitological investigation, 246, 169, 109, 94, and 92 were positive using TFT, MM, FEC, KK, and DWMM techniques, respectively. Overall, the prevalence of hookworm among patients in the study area was 63.23% (Table 1).

### 3.2. Performance of Hookworm Diagnosis Techniques Using CRS as the Gold Standard

Computation of the sensitivity of the laboratory diagnosis methods showed that the TFT had the highest sensitivity (100%). Moreover, its diagnostic accuracy was 100%. McMaster was the second both in terms of sensitivity (68.7%) and diagnostic accuracy (80%), followed by FEC and KK. The prevalence of hookworm using TFT and MM showed a statistically significant discrepancy with a difference rate of 20% (*p* < 0.001). The present finding also showed that DWMM had the lowest sensitivity (37.4%) and diagnostic accuracy (60%). All tests had 100% specificity and positive predictive values. TFT and MM had 100% and 65% NPVs, respectively, while the others had 48 to 51%. The sensitivity, specificity, diagnostic accuracy, and positive and negative predictive values are summarized in Table 2.

### 3.3. Performance of Hookworm Diagnosis Techniques Based on Infection Intensity

The mean intensities of infections were expressed as eggs per gram of stool (EPG). Out of the 246 hookworm-positive study participants, 77 (31.3%) and 92 (37.4%) were grouped under light and heavy infection categories, respectively, while the remaining 77 (31.3%) were not categorized to any of the infection intensity since they were only detected by the TFT. The sensitivity of DWMM, KK, and FEC techniques showed an increase in sensitivity as a function of increasing intensity of infection. On the other hand, the sensitivity of TFT and MM methods was not affected by the infection intensity. Amazingly, TFT detected a significant number of hookworm infections (a total of 77) which were not identified by the internationally accepted STH diagnosis tools (MM and KK techniques) (Table 3).

### 3.4. Degree of Agreement of Hookworm Diagnosis Techniques with CRS (Gold Standard)

The test tube flotation technique had a perfect agreement (agreement = 100%, kappa = 1) with CRS, followed by MM (moderate agreement = 80%, kappa = 0.67) and FEC (substantial agreement = 64%, kappa = 0.36). On the other hand, DWMM had a low degree of agreement (fair agreement = 60%, kappa = 0.30). The degree of agreement of hookworm diagnosis techniques with the composite reference standard (CRS) is summarized in Table 4.

## 4. Discussion

Ethiopia is one of the hotspot areas for hookworm and other STHs in the world [24–26]. Thus, integrated hookworm prevention and control measures are needed. Appropriate diagnosis is one of the most important tools in fighting the disease. It is recommended to use Kato-Katz, FEC, and McMaster methods for the detection of human soil-transmitted helminths (STHs) including hookworms. All of these and other techniques rely on visual examination of a small sample of stool to determine the presence and number of the parasitic ova with different sensitivities, especially in low transmission areas [27, 28]. It is clear that they are helpful in the disease diagnosis; however, they may not be equally sensitive and could also have their own limitations. For instance, DWMM is solely used in almost all health care facilities in Ethiopia and other developing countries due to its low cost and easy procedure. Nevertheless, it is undoubtedly known that the sensitivity of DWMM is poor [29, 30]. In spite of this, there are few studies that have evaluated the clinical sensitivity of DWMM compared to other microscopical techniques for the diagnosis of intestinal parasitosis including hookworm infection.

The present study evaluated the performance of five types of stool examination methods (TFT, MM, FEC, KK, and DWMM) for hookworm diagnosis using their composite reference standard as the gold standard method. It has been confirmed that DWMM has poor sensitivity. On the other hand, the test tube flotation technique was found to be more sensitive, cheaper, and easier to apply in the routine practices of hookworm identification than the others. It was reported that both the sensitivity and the diagnostic accuracy of this method were 100%. This is almost three times more sensitive than the commonly used DWMM (37%). The low sensitivity of DWMM might be related to the use of only a small amount of the stool sample (only 2 mg of stool is used in DWMM compared to about 4 g for the test tube flotation technique). Moreover, the presence of large stool debris materials in the DWMM may conceal the parasitic ova. The sensitivity of DWMM in this study (37%) is similar to other studies done in Ethiopia [29, 31]. This suggests that DWMM has resulted in around 63% false-negative reports during hookworm diagnosis. This substantially underestimates the prevalence of hookworm infection. Besides, similar to the current study, a study conducted in India showed that DWMM has a very low sensitivity for the detection of hookworm as compared with Kato-Katz and FEC [32]. Therefore, the use of the DWMM technique alone for the diagnosis of intestinal parasitic infection may not be appropriate and may result in misdiagnosis of intestinal parasitic infection. Moreover, it has a great impact on the control and elimination programs of hookworm and other soil-transmitted helminth infections. Thus, this study may encourage using TFT as a confirmatory test for hookworm infection in order to break the transmission cycle and ultimately reduce its morbidity and mortality.

The finding of the present study also demonstrated that MM is the second most sensitive test with a sensitivity and diagnostic accuracy of 68.7% and 80%, respectively. Even though it is one of the most recommended diagnosis methods for soil-transmitted helminths by the World Health Organization, its sensitivity is lower by about 33% compared to TFT in the current study. Formol-ether concentration and KK techniques were also ranked as the third (44.3%) and fourth (38.2%) sensitive tests, respectively, in this study. However, this is lower than the previous study carried out in Gondar in which their sensitivities were reported from 69 to 72.4% [31]. Similar to this study, a study carried out in India showed that the diagnostic sensitivity of FEC was high as compared to the KK technique [33]. This might be due to the disappearance of hookworm eggs due to glycerin following delays which occur between the time of KK smear preparation and microscopic examination. In addition to this, the observed differences in the sensitivities among different studies could be due to the infection intensity variation. Moreover, it might be related to the difference in the skill of the laboratory personnel. The observation of the lower sensitivity of MM, KK, and FEC than TFT from the current study indicates that another better diagnostic tool is necessary during patient diagnosis, monitoring, and evaluation of hookworm infection following the intervention.

According to the current study, in most of the stool examination methods, their sensitivity increases whenever more eggs are excreted in the stool. FEC, KK, and DWMM identified hookworm from only 22%, 2.6%, and 3.9% of the light infections, respectively. However, DWMM, KK, and FEC have detected the parasite from 97, 100, and 100 percent of moderately infected study participants, respectively. This may suggest that they will most likely not miss the moderate-to-heavy intensity of hookworm infections, which is mostly associated with morbidity [34, 35]. Hence, these three techniques will be able to diagnose those individuals who require treatment. Nonetheless, their inability to detect light infections properly may make them have an insignificant role in the evaluation of hookworm infection following MDA and other therapeutic efficacy studies. Thus, TFT might be taken as the best method over the other available diagnosis methods.

The specificity of all of the stool examination methods described in this study was 100%, and this is in line with findings of a study done in Gondar which revealed that DWMM, FEC, and KK had greater than 97% of sensitivity [31]. The current study also analyzed the level of agreement of the various types of hookworm diagnosis methods with the composite reference standard. The reproducibility of the TFT compared to CRS had a perfect agreement (agreement = 100%, kappa = 1). This indicates that the TFT is 100% as sensitive as the gold standard technique for the disease diagnosis. Next to TFT, McMaster showed a moderate agreement (agreement = 80%, kappa = 0.67), followed by formol-ether concentration and Kato-Katz techniques. Importantly, DWMM showed the lowest agreement (agreement = 60%, kappa = 0.30), and this may suggest its little role in the disease diagnosis. Generally, this study found an encouraging outcome and implies that TFT could be the most preferred technique for hookworm infection detection.

## 5. Limitation

There is no similar study conducted so far as TFT is concerned. As a result, this has made difficulties in making rigorous discussions on this finding.

## 6. Conclusion

The present study highlighted that the prevalence of hookworm infection is being underreported in Ethiopia due to the use of poor sensitive test methods. TFT is almost three and 1.5 times more sensitive in the diagnosis of hookworm infection than DWMM and McMaster techniques, respectively. It is also by far more sensitive than KK and FEC techniques. Apart from its better sensitivity, TFT is simple and does not require expensive materials. Thus, we recommend that laboratory professionals, who are found in hookworm endemic areas, should stick to TFT for its diagnosis. We also advocate that TFT has to be used as a major method of hookworm diagnosis during implementation and monitoring of mass drug administration.

## Figures and Tables

**Table 1:** Hookworm positivity rate by diagnosis techniques among the study participants.

Diagnosis tools	Hookworm infection	
	Number of positives, *n* (%)	Number of negatives, *n* (%)
TFT	246 (63.23)	143 (36.76)
MM	169 (43.44)	220 (56.55)
FEC	109 (28.02)	280 (71.97)
KK	94 (24.16)	295 (75.83)
DWMM	92 (23.65)	297 (76.34)
CRS	246 (63.23)	143 (36.76)

CRS: composite reference standard.

**Table 2:** Performance of hookworm diagnosis techniques compared to CRS.

Diagnosis tools	Sensitivity % (95% CI)	Specificity % (95% CI)	PPV % (95% CI)	NPV % (95% CI)	Diagnostic accuracy
TFT	100 (98, 100)	100 (97, 100)	100	100	100 (99, 100)
MM	68.7 (62, 74)	100 (97, 100)	100	65 (60, 69)	80 (75, 84)
FEC	44.3 (48, 50)	100 (97, 100)	100	51 (48, 54)	65 (60, 70)
KK	38.2 (32, 45)	100 (97, 100)	100	48 (46, 51)	61 (56, 66)
DWMM	37.4 (31, 44)	100 (97, 100)	100	48 (47, 51)	60 (55, 65)

**Table 3:** Performance of hookworm diagnosis techniques based on the infection intensity category.

Diagnosis tools	Number of positives, *n* (%)		
	Uncategorized infection intensity, *n* = 77	Light infection intensity, *n* = 77	Moderate infection intensity, *n* = 92
TFT	77	77 (100)	92 (100)
MM	0	77 (100)	92 (100)
FEC	0	17 (22)	92 (100)
KK	0	2 (2.6)	92 (100)
DWMM	0	3 (3.9)	89 (96.7)
CRS	77	77 (100)	92 (100)

**Table 4:** Degree of agreement of hookworm diagnosis techniques with the gold standard (CRS).

Diagnosis tools		CRS		Total	Agreement	Kappa value
		+	-			
TFT	+	246	0	246	100	1
	-	0	143	146		

MM	+	169	0	169	80.2	0.67
	-	77	143	220		

FEC	+	109	0	109	64.7	0.36
	-	137	143	280		

KK	+	94	0	94	61	0.31
	-	152	143	295		

DWMM	+	92	0	92	60	0.30
	-	154	143	297		

Kappa < 0: no agreement; 0.00-0.20: slight agreement; 0.21-0.40: fair agreement; 0.41-0.60: moderate agreement; 0.61-0.80: substantial agreement; 0.81-1.00: almost perfect agreement. CRS: composite reference standard.

## Data Availability

All data generated or analyzed during this study are included in this published article.

## Abbreviations

DWMM: Direct wet mount microscopy
EPG: Eggs per gram of stool
FN: False negative
FP: False positive
FEC: Formol-ether concentration
KK: Kato-Katz
MDA: Mass drug administration
MM: McMaster
NPV: Negative predictive value
NTD: Neglected tropical disease
PPV: Positive predictive value
STH: Soil-transmitted helminthiasis
TFT: Test tube flotation technique
TN: True negative
TP: True positive
WHO: World Health Organization.
